# Pharmacy Customers’ Experiences With Electronic Prescriptions: Cross-Sectional Survey on Nationwide Implementation in Finland

**DOI:** 10.2196/jmir.9367

**Published:** 2018-02-23

**Authors:** Elina Lämsä, Johanna Timonen, Riitta Ahonen

**Affiliations:** ^1^ School of Pharmacy Faculty of Health Sciences University of Eastern Finland Kuopio Finland

**Keywords:** electronic prescribing, pharmacies, patient satisfaction, surveys and questionnaires

## Abstract

**Background:**

One of the forerunners in electronic health, Finland has introduced electronic prescriptions (ePrescriptions) nationwide by law. This has led to significant changes for pharmacy customers. Despite the worldwide ambition to develop ePrescription services, there are few reports of nationally adopted systems and particularly on the experiences of pharmacy customers.

**Objective:**

The aim of this study was to investigate Finnish pharmacy customers’ (1) experiences with purchasing medicines with ePrescriptions; (2) experiences with renewing ePrescriptions and acting on behalf of someone else at the pharmacy; (3) ways in which customers keep up to date with their ePrescriptions; and (4) overall satisfaction with ePrescriptions.

**Methods:**

Questionnaires were distributed to 2913 pharmacy customers aged ≥18 years purchasing prescription medicines for themselves with an ePrescription in 18 community pharmacies across Finland in autumn 2015. Customers’ experiences were explored with 10 structured questions. The data were stored in SPSS for Windows and subjected to descriptive analysis, chi-square, Fisher exact, Kolmogorov-Smirnov, the Mann-Whitney U, and Kruskal-Wallis tests.

**Results:**

Completed questionnaires were returned by 1288 customers, a response rate of 44.19% (1288/2913). The majority of the respondents did not encounter any problems during pharmacy visits (1161/1278, 90.85%) and were informed about the current status of their ePrescriptions after their medication was dispensed (1013/1276, 79.44%). Over half of the respondents had usually received a patient instruction sheet from their physician (752/1255, 59.92%), and nearly all of them regarded its content as clear (711/724, 98.2%). Half of the respondents had renewed their ePrescriptions through the pharmacy (645/1281, 50.35%), and one-third of them had acted on behalf of someone else with ePrescriptions (432/1280, 33.75%). Problems were rarely encountered in the renewal process (49/628, 7.8%) or when acting on behalf of another person (25/418, 6.0%) at the pharmacy. The most common way of keeping up to date with ePrescriptions was to ask at the pharmacy (631/1278, 49.37%). The vast majority of the respondents were satisfied with ePrescriptions as a whole (1221/1274, 95.84%).

**Conclusions:**

Finnish pharmacy customers are satisfied with the recently implemented nationwide ePrescription system. They seldom have any difficulties purchasing medicines, renewing their ePrescriptions, or acting on behalf of someone else at the pharmacy. Customers usually keep up to date with their ePrescriptions by asking at the pharmacy. However, some customers are unaware of the practices or have difficulty keeping up to date with the status of their ePrescriptions. The provision of relevant information and assistance by health care professionals is therefore required to promote customers’ adoption of the ePrescription system.

## Introduction

### Introduction of ePrescriptions

Electronic health (eHealth), including electronic health records, electronic identification, online portals, and mobile health apps, has rapidly become common not only among health care providers but also patients and consumers. eHealth is about efficient and high-quality services, empowerment of patients, as well as enhanced information exchange and communication between health care professionals and patients [1]. As part of eHealth strategies, the implementation of electronic prescriptions (ePrescriptions) has been assessed as a pharmaceutical policy reform in many countries [2-4]. For example, in 2017, the Government of Canada invested Can $300 million over 5 years to enhance eHealth activities, including expanding ePrescribing and patients’ access to their electronic health records [5]. The implementation of ePrescriptions primarily aims not just to improve medication safety but also to enhance the prescribing, dispensing, and purchasing of medicines [2,6]. The definition of ePrescription, however, varies between electronic transfer of a prescription from a prescriber to a particular pharmacy and a nationwide system where prescriptions are issued, transferred, stored, dispensed, and renewed via an information network [2,4,6]. This latter system has so far been introduced in a few countries including Sweden, Estonia, Denmark, the Netherlands, and Finland.

Once introduced, health care policy reforms should be rigorously evaluated by comparing their actual impacts with those anticipated before implementation [7,8]. The evaluation should encompass the effects from different viewpoints. The implementation of ePrescriptions has been widely studied from the perspective of health care professionals. Studies have concerned topics such as the benefits and problems of ePrescriptions [9-11], facilitators and barriers to their implementation [12], and their impact on workflow and medication safety [11,13-15]. However, experiences from nationwide ePrescription systems have been reported in only a few studies [7,9,14-17].

### Patients’ Perceptions of ePrescriptions

Despite researchers’ wide interest in the implementation of ePrescriptions, the literature on the experiences of patients—one of the main user groups—is limited. The aim of ePrescriptions is to offer enhanced health care for patients. They enable the electronic storing of prescriptions and, as a consequence, streamline the purchasing of medicines at the pharmacy or even online [17]. Furthermore, electronic storing of medical records has provided patients with access to their personal records via Web-based services, and this has given them a more active role in the management of their medications [18-20]. It is therefore important to study whether patients have sufficient understanding of the system, whether they know how to use the services, and whether the services operate properly [21].

Patients’ perceptions of ePrescriptions have been studied in the United States [22-26] and Sweden [17]. Studies conducted in Australia [27] and Scotland [28] have reported patients’ attitudes toward ePrescriptions before the implementation. However, the study settings have mainly been local and the samples rather small, involving patients from 1 clinic [23,24,27] or 1 state [25,26]. According to the literature, patients’ attitudes toward ePrescriptions are positive [17,22,23,27,28] partly as a result of their experiences with ePrescription services [17,22]. An ePrescription has commonly been defined as a prescription that a prescriber electronically issues and sends to a certain pharmacy [22-27]. To the authors’ knowledge, patients’ attitudes toward a nationwide ePrescription system have only been studied in Sweden [17]. However, there is little published information on how the nationwide system works in practice from the patient’s perspective and how patients keep up to date with their ePrescriptions.

This study is part of a research project studying health care professionals’ and patients’ experiences with the recently implemented nationwide ePrescription system in Finland. The project aims to investigate whether the system has achieved the objectives predefined by law. The objective of this study was to investigate Finnish pharmacy customers’ experiences with ePrescriptions. The specific aims were to explore the following:

- experiences of purchasing medicines with ePrescriptions
- experiences of renewing ePrescriptions and acting on behalf of someone else at the pharmacy
- how customers keep up to date with their ePrescriptions
- how satisfied they are with the ePrescription system as a whole

## Methods

### Study Context

#### Introduction of ePrescriptions in Finland

In Finland, the nationwide introduction of ePrescriptions by law began in 2012, and Finnish pharmacies started to dispense ePrescriptions in the same year [29]. The issuing of ePrescriptions was made obligatory in public health care in 2013 and in private health care in 2015. At the beginning of 2017, all health care providers were obliged to introduce the ePrescription system, and nowadays, conventional prescriptions (eg, paper, telephone) are only permitted in exceptional situations such as blackouts. The use of ePrescriptions aims to improve patient and medication safety as well as facilitate and streamline the prescribing and dispensing of medicines. In 2015, when the survey reported in this paper was conducted, over 90% of nearly 56 million prescriptions dispensed in Finnish pharmacies were electronic [30,31].

#### The ePrescription System From the Patient’s Perspective

In Finland, ePrescriptions are issued, transferred, stored, and dispensed electronically [29]. Once issued, ePrescriptions are stored in a centralized database (the Prescription Centre) from which they can be retrieved and dispensed in any Finnish pharmacy. The patient can choose the most convenient pharmacy at the time of purchasing the medicine. Patients can view their ePrescriptions and other personal medical records in a Web service called My Kanta [19,32].

A patient who is present when an ePrescription is issued is entitled to receive a patient instruction sheet from the physician. The document includes a summary of the ePrescription: the brand or generic name of the medicine prescribed, dosage instructions, prescriber, place and date of prescribing, quantity or duration of therapy, and the expiry date of the prescription. However, the patient can decline to receive a patient instruction sheet.

At the pharmacy, a patient gets his or her medicines dispensed by showing a patient instruction sheet, a personal health insurance card, or other valid ID [33]. The Act on Electronic Prescriptions 61/2007 requires the pharmacist to give the patient the latest information about the amount of medication still to be dispensed. The information is also printed out onto the dispensing label to be attached to the medicine package.

The patient can ask for a renewal of an ePrescription at a health care unit or at a pharmacy from where the renewal request is sent electronically to a health care unit [33]. Patients can ask someone else to act on their behalf at the pharmacy. The person purchasing another’s medicine with an ePrescription is required to present the patient’s insurance card or a patient instruction sheet. Signed consent is needed when a patient authorizes another person to ask for a renewal of an ePrescription, requests a printed summary of his or her ePrescriptions, or asks the pharmacist, physician, or nurse to delete his or her ePrescription.

#### Validity of Prescriptions

In Finland, a prescription is valid for 2 years from the date of issue, the exceptions being prescriptions for central nervous system agents with abuse potential or narcotic agents, which are valid for 12 months. Prescription validity was extended from 12 months to 24 months in January 2017; so, at the time of this study, ePrescriptions were valid for 1 year. A prescription can be prescribed for a specific period (*1-year dosage*) or for an amount (*400 tablets*). Nevertheless, patients can be reimbursed for the maximum of 3 months’ dosage dispensed at the pharmacy at a time. Consequently, a reimbursable purchase for 3 months’ medication can be repeated 4 times during the validity period of 1 year.

### The Survey

A questionnaire survey was conducted among pharmacy customers aged ≥18 years who were purchasing medicines for themselves with ePrescriptions in autumn 2015. Questionnaires were handed out by 18 different-sized pharmacies across Finland. The number of questionnaires delivered to each pharmacy varied between 30 and 200, and was adjusted according to the number of prescriptions dispensed daily at the pharmacy. We provided pharmaceutical staff with instructions on the distribution of questionnaires. Pharmacists informed customers about the study after dispensing their medication and offered them the questionnaire together with a franked envelope for their responses. They were not required to keep a list of customers who declined to participate. The questionnaires were handed out as long as there were forms left, but for a maximum of 2 weeks. After the study period, pharmacies reported the number of questionnaires left to compute the response rate. Altogether, 2915 questionnaires were distributed. Reminders could not be sent as we were unaware of the customers’ addresses.

### The Questionnaire

The 4-page form contained 26 questions (Multimedia Appendix 1). The main themes of the questions were as follows: (1) customers’ experiences with purchasing medicines with ePrescriptions, (2) experiences with and opinions on the My Kanta service, (3) opinions on the benefits and problems with ePrescriptions, and (4) information sources and information needs related to ePrescriptions. The questionnaire was designed on the basis of the mandated objectives of ePrescriptions [29], the anticipated impacts of ePrescriptions [34], and some previous studies [17,35]. The questionnaire was pilot tested in a local pharmacy in spring 2015. The pilot customers were interviewed at the pharmacy after filling in the questionnaires to check that they had understood the questions. Minor modifications were made based on the pilot test. However, modifying the questions reported in this paper was not necessary.

This paper reports results from 10 questions related to respondents’ experiences with purchasing medicines with ePrescriptions, renewing ePrescriptions through the pharmacy, acting on behalf of another person, and their overall satisfaction with ePrescriptions (Questions 2-10 and 21 in Multimedia Appendix 1). Two questions were defined with the phrase “this time at the pharmacy,” the aim being to gain cross-sectional data about technical problems or ambiguities in ePrescriptions during the dispensing process and how pharmacists comply with the instruction to inform customers about their ePrescription details. All the questions were structured. Some questions had space for respondents to expand on their answer, and some were multiple choice questions. The question asking how satisfied the respondent is with ePrescriptions as a whole was answered on a 6-point Likert-type rating scale, where 1 represented not at all satisfied and 6 very satisfied.

Structured questions yielded background information on the respondents’ gender, area of residence, education, and regularity of prescription medicine use (Questions 22 and 24-26 in Multimedia Appendix 1). The respondent’s year of birth was obtained by means of an open-ended question (Question 23 in Multimedia Appendix 1). Furthermore, to explore the extent of respondents’ experiences, a structured question asked respondents to estimate how many times they had purchased medicines with ePrescriptions within the last 6 months (Question 1 in Multimedia Appendix 1).

### Statistical Methods

Data from the questionnaires were entered into the Statistical Package for Social Scientists software (version 23.0 SPSS Inc, Chicago, IL, USA). A descriptive approach (frequencies, means) was used in the analysis. In the analyses, the respondent’s age was placed into 1 of the 4 groups (18-34, 35-59, 60-74, and 75 years or older). Categorical data from questions concerning respondents’ experiences with ePrescriptions, renewals at the pharmacy, acting on behalf of someone else, and checking the status of ePrescription were compared with background variables using either the Pearson chi-square test or two-sided Fisher exact test. After Kolmogorov-Smirnov normality test, statistical significance of differences between means in independent groups was assessed using the Mann-Whitney *U* or Kruskal-Wallis tests for the satisfaction with ePrescriptions. Statistical significance was determined as *P*<.05.

### Ethical Statement

The study setting and research process complied with local and national ethical instructions for research [36]. This study required no ethical approval.

## Results

### Study Population

In total, 2915 questionnaires were handed out to pharmacy customers and 1290 were returned to the authors. Two forms, however, were excluded as the respondents were aged less than 18 years. Consequently, the study sample was 2913, giving a response rate of 44.21% (N=1288).

Background information on the study population is presented in Table 1.

**Table 1:** Characteristics of the study respondents (N=1288).

Characteristic		n (%)
**Gender (n=1287**^a^**)**		
	Female	965 (74.98)
	Male	322 (25.02)
**Age in years (n=1167**^a^**)**		
	18-34	137 (11.74)
	35-59	379 (32.48)
	60-74	476 (40.79)
	≥75	175 (15.00)
**Education (n=1263**^a^**)**		
	Basic education (comprehensive school)	274 (21.69)
	Vocational degree	459 (36.34)
	Secondary school graduate	152 (12.03)
	Lower-level university degree	203 (16.07)
	Higher-level university degree	175 (13.86)
**Current use of prescription medicines (n=1272**^a^**)**		
	Temporarily	117 (9.20)
	Regularly	715 (56.21)
	Both regularly and temporarily	440 (34.59)
**Area of residence (n=1276**^a^**)**		
	Southern Finland	301 (23.59)
	Southwestern Finland	208 (16.30)
	Western and Central Finland	205 (16.07)
	Eastern Finland	183 (14.34)
	Northern Finland	256 (20.06)
	Lapland	123 (9.64)
**Medicine purchases with an ePrescription within the last six months (N=1283**^a^**)**		
	First time during the study	37 (2.88)
	2-5 times	688 (53.62)
	6-10 times	335 (26.11)
	Over 10 times	223 (17.38)

^a^Some of the respondents did not report their gender, age, education, usage of prescription medicines, area of residence, or how many times they had purchased medicines with an ePrescriptions within the last 6 months.

The majority of the respondents were female and the mean age was 59 years (range 18-93, median 62). All 6 geographical areas of Finland were represented among the study population. Most of the respondents had gained experience with purchasing medicines with ePrescriptions several times within the previous 6 months.

### Experiences With the Patient Instruction Sheet

Over half (752/1255, 59.92%) of the respondents had usually received a patient information sheet concerning the prescribed medicine from the physician (Table 2). Over one-third of the respondents (503/1255, 40.08%) reported not receiving the information sheet: for 23.75% (298/1255), the physician did not offer it, whereas 5.58% (70/1255) reported they had declined to receive it. The rest (135/1255, 10.76%) did not know what a patient information sheet is. Those who had usually received the sheet generally regarded its content as clear (711/724, 98.2%). However, respondents in the age group 75 years and older were likely to state that the content was unclear (*P*=.04).

### Experiences With Purchasing Medicines With ePrescriptions

The majority (1161/1278, 90.85%) of the respondents reported not encountering any problems with their ePrescriptions at the pharmacy (Table 2). Almost one-tenth (117/1278), however, experienced some inconvenience or a problem during the visit.

From the total of 162 reported problems, the most common were that the ePrescription had expired without the respondent’s knowledge (59/117, 50.4%), the ePrescription had no medication remaining and the respondent was unaware of this (40/117, 34.2%), and the physician had not sent the ePrescription as promised (22/117, 18.8%). Those aged 75 years and older and respondents using prescription medicines both regularly and temporarily were more likely to experience problems (*P*=.005 and *P*=.04, respectively) compared with others (Table 2).

Most of the respondents (1013/1276, 79.29%) were told how much medication was remaining on their ePrescription after the purchase (Table 2). Older pharmacy customers were more likely to be informed about the number of batches remaining than working age customers (*P*<.001). Furthermore, significant differences were observed between respondents’ education level (*P*=.02) and the regularity of prescription medicine use (*P*<.001). Those with higher levels of education and those using prescription medicines only temporarily were unlikely to be informed compared with respondents with only basic education or using prescription medicines regularly.

### Experiences With ePrescription Renewals and Acting on Behalf of Someone Else

Approximately half of the respondents (645/1281, 50.35%) had renewed their ePrescriptions through the pharmacy (Table 2). Significant differences were observed between genders (*P*=.033), age groups (*P*<.001), education (*P*<.001), regularity of prescription medicine use (*P*<.001), and area of residence (*P*<.001). Those who had made the renewal request at the pharmacy rarely encountered any problems during the process (579/628, 92.2%). However, 49 respondents (7.8%) reported inconveniences such as long waiting times, medication running out before the renewal, technical problems, no notification that a renewal had been authorized or that the ePrescription had not been renewed despite the customer’s request.

One-third of the respondents (432/1280, 33.75%) had acted on behalf of someone else with an ePrescription at the pharmacy. The majority of those who had acted on behalf of others were satisfied with the service (393/418, 94.1%), whereas 25 respondents (6%) reported problems such as being unaware of the required signed consent or a parent’s inability to access a minor’s up-to-date ePrescription details in the My Kanta service.

### Experiences With Keeping Up to Date With ePrescriptions

The respondents generally kept up to date with their ePrescription status (eg, amount of medication remaining or expiry date of a prescription) by asking at the pharmacy (631/1278, 49.37%), reading the label affixed to the medication package (574/1278, 44.91%), or using the My Kanta Web service (491/1278, 38.42%); see Table 3.

Asking at the pharmacy and reading the dispensing label were the most common methods among the oldest age group (*P*=.01 and *P*<.001, respectively). In contrast, the oldest pharmacy customers were less likely to use My Kanta than younger respondents (*P*<.001).

Reading the dispensing label was also common among women (*P*=.008), respondents using prescription medicines regularly (*P*<.001), and respondents with only basic education (*P*<.001). The My Kanta service was used regularly by respondents with education higher than comprehensive school (*P*<.001) and by those using prescription medicines both regularly and temporarily (*P*=.03).

### Overall Satisfaction With ePrescriptions

The general attitude toward ePrescriptions was positive among the respondents. On the 6-point Likert scale, 95.84% (1221/1274) of all respondents rated their overall satisfaction as 4 to 6 (Table 4). The mean for the level of satisfaction was 5.35. Overall satisfaction differed significantly between age groups (*P*=.049), respondents’ education (*P*<.001), and regularity of prescription medicine use (*P*=.03). The oldest respondents, respondents with only basic education, and respondents using prescription medicines regularly were likely to be very satisfied with ePrescriptions.

**Table 2:** Respondents’ experiences with ePrescriptions.

Characteristic		Has usually received a patient instruction sheet from a physician	Perceives the content of the patient instruction sheet as clear^a^	Did not encounter any problems with his or her ePrescription(s) at the pharmacy	Was told about the amount of medication still to be dispensed under the ePrescription(s) at the pharmacy	Has renewed ePrescription(s) through the pharmacy
All, n (%)		752 (59.92)	711 (98.20)	1161 (90.85)	1013 (79.39)	645 (50.35)
**Gender**		*P*=.42	*P*=.74	*P*=.47	*P*=.10	*P*=.03
	Female, n (%)	571 (60.6)	542 (98.0)	867 (90.5)	751 (78.3)	466 (48.6)
	Male, n (%)	181 (58.0)	169 (98.8)	293 (91.8)	261 (82.6)	178 (55.5)
**Age in years**		*P*=.545	*P*=.039	*P*=.005	*P*<.001	*P*<.001
	18-34, n (%)	77 (56.2)	71 (97)	127 (92.7)	96 (70.1)	30 (21.9)
	35-59, n (%)	224 (59.3)	215 (99.1)	354 (93.7)	274 (73.3)	144 (38.2)
	60-74, n (%)	283 (61.8)	271 (98.9)	425 (90.0)	397 (84.5)	282 (59.6)
	≥75, n (%)	96 (56.8)	86 (95)	146 (84.4)	150 (85.7)	132 (75.9)
**Education**		*P*=.73	*P*=.06	*P*=.20	*P*=.02	*P*<.001
	Basic education, n (%)	151 (59.5)	144 (99.3)	238 (87.5)	229 (85.1)	182 (67.4)
	Vocational degree, n (%)	277 (61.4)	265 (99.3)	415 (91.0)	365 (80.2)	251 (54.9)
	Secondary school graduate, n (%)	94 (62.7)	90 (99)	142 (93.4)	117 (77.0)	56 (36.8)
	Lower-level university degree, n (%)	117 (57.9)	106 (95.5)	182 (91.0)	147 (72.8)	73 (36.1)
	Higher-level university degree, n (%)	98 (56.6)	94 (96.9)	163 (93.1)	133 (76.9)	65 (37.1)
**Current use of prescription medicines**		*P*=.16	*P*=.13	*P*=.04	*P*<.001	*P*<.001
	Temporarily, n (%)	69 (59.0)	68 (100)	111 (94.9)	74 (64.3)	30 (25.6)
	Regularly, n (%)	399 (57.9)	377 (99.0)	650 (91.9)	570 (80.6)	376 (52.7)
	Both regularly and temporarily, n (%)	278 (63.6)	261 (97.0)	387 (88.4)	354 (80.8)	228 (52.3)
**Area of residence**		*P*=.07	*P*=.56	*P*=.17	*P*=.29	*P*<.001
	Southern Finland, n (%)	174 (59.6)	161 (97.0)	275 (91.7)	232 (77.6)	110 (36.8)
	Southwestern Finland, n (%)	132 (64.1)	122 (98.4)	193 (92.8)	153 (74.3)	94 (45.4)
	Western and Central Finland, n (%)	135 (67.5)	129 (98.5)	183 (91.0)	164 (80.8)	107 (52.7)
	Eastern Finland, n (%)	100 (56.2)	94 (96.9)	162 (89.5)	145 (79.7)	115 (63.2)
	Northern Finland, n (%)	136 (54.6)	131 (99.2)	223 (87.5)	208 (82.2)	130 (51.0)
	Lapland, n (%)	70 (58.3)	69 (100)	115 (95.0)	101 (82.8)	80 (65.0)

^a^Respondents who had usually received a patient information sheet answered the question.

**Table 3:** How respondents (N=1278) check the status of their ePrescriptions (eg, amount of medication remaining or expiry date of a prescription). A respondent may have chosen several alternatives.

Characteristic		Ask at the pharmacy	Read the label affixed to the medication package	Use the My Kanta service	Keep track of it themselves	Read the patient instruction sheet	Do not check it at all	Use another method^a^
All, n (%)		631 (49.37)	574 (44.91)	491 (38.42)	109 (8.53)	72 (5.63)	65 (5.09)	25 (1.96)
**Gender**		*P*=.13	*P*=.008	*P*=.42	*P*=.32	*P*=.40	*P*> *.99*	*P*=.13
	Female, n (%)	485 (50.3)	451 (46.7)	362 (37.5)	86 (8.9)	57 (5.9)	48 (5.0)	22 (2.3)
	Male, n (%)	146 (45.3)	123 (38.2)	129 (40.1)	23 (7.1)	15 (4.7)	16 (5.0)	3 (0.9)
**Age in years**		*P*=.01	*P*<.001	*P*<.001	*P*=.97	*P*=.02	*P*=.002	*P*=.40
	18-34, n (%)	75 (54.7)	50 (36.5)	65 (47.4)	11 (8.0)	10 (7.3)	13 (9.5)	5 (3.6)
	35-59, n (%)	187 (49.3)	140 (36.9)	180 (47.5)	29 (7.7)	23 (6.1)	26 (6.9)	6 (1.6)
	60-74, n (%)	208 (43.7)	245 (51.5)	183 (38.4)	40 (8.4)	15 (3.2)	15 (3.2)	10 (2.1)
	≥75, n (%)	98 (56.0)	98 (56.0)	26 (14.9)	13 (7.4)	15 (8.6)	4 (2.3)	2 (1.1)
**Education**		*P*=.24	*P*<.001	*P*<.001	*P*=.047	*P*=.04	*P*<.001	*P*=.31
	Basic education, n (%)	130 (47.4)	147 (53.6)	68 (24.8)	20 (7.3)	8 (2.9)	10 (3.6)	5 (1.8)
	Vocational degree, n (%)	221 (48.1)	221 (48.1)	187 (40.7)	34 (7.4)	22 (4.8)	17 (3.7)	7 (1.5)
	Secondary school graduate, n (%)	81 (53.3)	67 (44.1)	62 (40.8)	23 (15.1)	11 (7.2)	3 (2.0)	1 (0.7)
	Lower-level university degree, n (%)	110 (54.2)	69 (34.0)	94 (46.3)	17 (8.4)	14 (6.9)	14 (6.9)	6 (3.0)
	Higher-level university degree, n (%)	77 (44.0)	56 (32.0)	72 (41.1)	15 (8.6)	16 (9.1)	21 (12.0)	6 (3.4)
**Current use of prescription medicines**		*P*=.07	*P*<.001	*P*=.03	*P*=.74	*P*<.001	*P*<.001	*P*=.18
	Temporarily, n (%)	51 (43.6)	34 (29.1)	40 (34.2)	12 (10.3)	12 (10.3)	22 (18.8)	2 (1.7)
	Regularly, n (%)	337 (47.1)	317 (44.3)	257 (35.9)	58 (8.1)	23 (3.2)	31 (4.3)	10 (1.4)
	Both regularly and temporarily, n (%)	234 (53.2)	217 (49.3)	191 (43.4)	37 (8.4)	37 (8.4)	11 (2.5)	13 (3.0)
**Area of residence**		*P*=.20	*P*=.69	*P*=.97	*P*=.10	*P*=.07	*P*=.72	*P*=.26
	Southern Finland, n (%)	142 (47.2)	130 (43.2)	118 (39.2)	23 (7.6)	18 (6.0)	16 (5.3)	9 (3.0)
	Southwestern Finland, n (%)	93 (44.7)	91 (43.8)	80 (38.5)	24 (11.5)	20 (9.6)	12 (5.8)	3 (1.4)
	Western and Central Finland, n (%)	92 (44.9)	100 (48.8)	75 (36.6)	17 (8.3)	12 (5.9)	10 (4.9)	4 (2.0)
	Eastern Finland, n (%)	101 (55.2)	86 (47.0)	69 (37.7)	11 (6.0)	6 (3.3)	7 (3.8)	4 (2.2)
	Northern Finland, n (%)	133 (52.0)	107 (41.8)	103 (40.2)	28 (10.9)	9 (3.5)	10 (3.9)	1 (.4)
	Lapland, n (%)	63 (51.2)	53 (43.1)	45 (36.6)	5 (4.1)	7 (5.7)	9 (5.0)	3 (2.4)

^a^Freely worded answers included “ask the physician about it,” “use a Web portal of a private health center,” and “monitor the consumption of the medication.”

**Table 4:** Respondents’ (N=1274) overall satisfaction with ePrescriptions on a 6-point Likert scale, from 1 being "not at all satisfied" to 6 being "very satisfied."

Characteristic		1, n (%)	2, n (%)	3, n (%)	4, n (%)	5, n (%)	6, n (%)	*P* value
All		13 (1.0)	12 (0.9)	28 (2.2)	89 (7.0)	447 (35.1)	685 (53.8)	
**Gender**								.55
	Female	10 (1.0)	5 (1.6)	2 (0.6)	24 (7.6)	107 (34.0)	174 (55.2)	
	Male	3 (1.0)	7 (0.7)	26 (2.7)	65 (6.8)	339 (35.4)	511 (53.3)	
**Age in years**								.049
	18-34	0 (0.0)	1 (0.7)	4 (2.9)	8 (5.9)	60 (44.1)	63 (46.3)	
	35-59	2 (0.5)	3 (0.8)	4 (1.1)	23 (6.1)	157 (41.8)	187 (49.7)	
	60-74	4 (0.8)	4 (0.8)	11 (2.3)	41 (8.7)	138 (29.2)	275 (58.1)	
	≥75	6 (3.6)	3 (1.8)	4 (2.4)	5 (3.0)	45 (26.6)	106 (62.7)	
**Education**								<.001
	Basic education	6 (2.2)	1 (0.4)	6 (2.2)	14 (5.2)	63 (23.5)	178 (66.4)	
	Vocational degree	4 (0.9)	7 (1.5)	10 (2.2)	36 (7.9)	154 (33.8)	244 (53.6)	
	Secondary school graduate	1 (0.7)	0 (0)	5 (3.3)	15 (10.0)	60 (40.0)	69 (46.0)	
	Lower-level university degree	1 (0.5)	2 (1.0)	4 (2.0)	4 (2.0)	86 (42.6)	105 (52.0)	
	Higher-level university degree	1 (0.6)	1 (0.6)	2 (1.1)	18 (10.3)	79 (45.1)	74 (42.3)	
**Current use of prescription medicines**								.03
	Temporarily	0 (0)	1 (0.9)	3 (2.6)	11 (9.4)	47 (40.2)	55 (47.0)	
	Regularly	8 (1.1)	3 (0.4)	11 (1.6)	43 (6.3)	240 (34.0)	400 (56.7)	
	Both regularly and temporarily	5 (1.1)	7 (1.6)	11 (2.5)	35 (8.0)	156 (35.8)	222 (50.9)	
**Area of residence**								.73
	Southern Finland	4 (1.3)	4 (1.3)	7 (2.3)	26 (8.7)	95 (31.9)	162 (54.4)	
	Southwestern Finland	0 (0)	1 (0.5)	5 (2.4)	16 (7.7)	86 (41.5)	99 (47.8)	
	Western and Central Finland	2 (1.0)	3 (1.5)	6 (3.0)	10 (5.0)	69 (34.5)	110 (55.0)	
	Eastern Finland	2 (1.1)	1 (0.5)	2 (1.1)	10 (5.5)	67 (36.8)	100 (54.9)	
	Northern Finland	2 (0.8)	2 (0.8)	7 (2.8)	14 (5.5)	88 (34.8)	140 (55.3)	
	Lapland	2 (1.6)	1 (0.8)	1 (0.8)	12 (9.8)	41 (33.6)	65 (53.3)	

## Discussion

### Principal Findings and Comparison With Prior Work

This study found that Finnish pharmacy customers are highly satisfied with ePrescriptions as a whole. They rarely encounter any problems in purchasing medicines with ePrescriptions, in acting on behalf of someone else, or in renewing ePrescriptions at the pharmacy. The most common way of keeping up to date with their ePrescriptions is to ask at the pharmacy. The results are in line both with the literature [17,23,26] and with our earlier studies [19,37].

Finnish pharmacy customers are very satisfied with the nationwide ePrescription system, even more than the Swedes [17] or pharmacists and physicians in Finland [14,15]. In our earlier study, pharmacy customers reported benefits more often than problems in ePrescriptions [37]. Overall, the most common benefits of using ePrescriptions highlighted by medicine users are the ease of purchasing prescription medicines and the electronic storing of prescriptions [17,22,23,37].

Despite its several benefits, Finnish health care professionals have criticized the ePrescription system as slow and rigid [14,15]. Although the new system has streamlined the dispensing process as a whole, many community pharmacists reported facing errors or ambiguities in ePrescriptions requiring clarification during the dispensing process every week or even daily [15]. However, the results of this study indicate that pharmacy customers are not conscious of the rigidity of data systems or defects in ePrescriptions.

Many problems customers encountered during the pharmacy visit were related to their lack of awareness of the current status of their ePrescriptions such as the expiry date and to insufficient information received about the services. The consequences of these problems remain unknown, but they may have caused a gap in the customer’s medication or at least a new visit to the pharmacy. Customers’ difficulty keeping up to date with their ePrescribed medications has also been reported in our earlier studies among Finnish pharmacy staff and pharmacy customers [37,38] as well as in studies conducted in the United States [22-24,39]. In Finland, the difficulty occurs particularly among older persons and medicine users without a computer, the Internet, or electronic IDs, which are required to log into the My Kanta service [37,38]. According to this study, most of the respondents check the status of their ePrescriptions by asking at the pharmacy, whereas the My Kanta service was used more rarely. In addition to inaccessibility, one reason why the Web service is relatively little used could be that customers are unaware of it [19]. Medicine users’ familiarity with the Web service should be enhanced as eHealth services aim to improve patient empowerment. However, there will still be patients refusing or being unable to use new technologies. Future studies should examine which existing or new methods they prefer to receive information about their ePrescriptions.

Another finding suggesting pharmacy customers need assistance or additional information on ePrescription practices was that several respondents were unfamiliar with the patient instruction sheet. According to the Finnish Act on Electronic Prescriptions, the health care units involved are required to provide information about ePrescriptions and related services for patients before they receive an ePrescription for the first time [29]. However, laypeople probably need to be given information several times before they can adopt the new technology, which implies that the obligation to provide information should be extended from prescribers to dispensers. The information needs to be consistent and offered not only on the first occasion but repeatedly to new users of the ePrescription service. The health care professionals involved must also be properly trained in the new services in advance.

Some of the problems reported in renewing ePrescriptions and acting on behalf of someone else at the pharmacy may now be outdated because the system has undergone a few improvements since the survey was conducted. For one thing, patients have since been able to use the My Kanta service to send renewal requests by themselves. A phone number can be added to the request for a text message stating when a renewal has been authorized. Not only is the process convenient and streamlined but the electronic renewal of prescriptions via Web-based portals may also improve patients’ medication adherence and the results of medication therapies [20,40,41]. Second, guardians are now entitled to view the ePrescriptions and other health records of their dependents less than 10 years in My Kanta. In the future, it would be sensible to allow patients to consent to another person acting on their behalf via the My Kanta service. The paperless system would ease the workload on pharmacies and health care units, which are required to archive consents for 12 years after they have expired. In addition, an electronic consent would be easier for the patient to cancel or change. To promote the use of My Kanta, a mobile app could be one of the future developments.

### Strengths and Limitations

The findings of this study are based on a fully operational ePrescription system that had been in nationwide use for a few years during the survey. Most of the respondents had purchased medicines with an ePrescription several times before the study. The method used prevented us from sending out reminders but was suitable for reaching the target population, that is, persons with experiences of ePrescriptions. The response rate of 44% is comparable with that of other studies conducted with similar methods [42,43]. As the questionnaires were randomly distributed by pharmacists, we had no background information on the customers asked to participate in the study. Comparable statistics on Finnish pharmacy customers’ characteristics are not available. However, the respondents’ use of prescription medicines (regularly, temporarily, or both) as well as age and gender distributions were similar to those of an earlier study surveying Finnish pharmacy customers’ experiences with generic substitution conducted with a similar method [42]. Consequently, we believe our study population well represents those customers purchasing their prescription medicines at Finnish community pharmacies.

This study explored Finnish pharmacy customers’ first experiences with the recently implemented ePrescriptions in 2015. The nationwide system has been further developed ever since. To evaluate patients’ adaptation to the ePrescription system more rigorously, their experiences, overall satisfaction, and ways of keeping up to date with their ePrescriptions should be continuously explored as the system matures. On the other hand, while the digital divide constantly narrows, future studies should investigate whether the new digital generation has different perspectives and ideas on how to make the ePrescription system more customer friendly.

### Conclusions

Overall, Finnish pharmacy customers are highly satisfied with ePrescriptions and rarely have any problems with them. The problems encountered are often related to customers’ unawareness of the practices and difficulty keeping up to date with the status of their ePrescriptions. Despite the national Web service for viewing ePrescriptions, customers’ most common way of keeping up to date with their ePrescriptions is to ask at the pharmacy. In conclusion, pharmacy customers need clear information and assistance from health care professionals in advance and during implementation of a nationwide ePrescription system.

## Appendix

Multimedia Appendix 1 The Questionnaire.

## Abbreviations

eHealth: electronic health
ePrescriptions: electronic prescriptions
