# Supplementary material for: Pharmacy Customers’ Experiences With Electronic Prescriptions: Cross-Sectional Survey on Nationwide Implementation in Finland
Source: J Med Internet Res. 2018 Feb 23;20(2):e68. doi: 10.2196/jmir.9367 (PMC5845104; doi:10.2196/jmir.9367)
Supplement: Multimedia Appendix 1 [file jmir_v20i2e68_app1.pdf]

University of Eastern Finland
Faculty of Health Sciences
School of Pharmacy
Autumn 2015

Questionnaire study for pharmacy customers regarding electronic prescriptions (ePrescriptions)

Answer the questions by circling the number of the most suitable alternative. If necessary, write the answer or the reason for the answer in the space provided. It is important for the study that you answer all the questions.

1. Estimate how many times you have purchased medication with an ePrescription <u>in the last six months</u>

1 This was the first time

2 2–5 times

3 6–10 times

4 More than 10 times

2. Did you have any problems with your ePrescription(s) <u>this time at the pharmacy</u>?

1 No
2 Yes. What kind? You may choose several alternatives if you had problems with several ePrescriptions.

   a. The physician had not sent the ePrescription as promised
   b. The ePrescription had expired without my knowledge
   c. The ePrescription had no medication remaining and I didn't know this
   d. The ePrescription was erroneous or lacked information, so the pharmacist had to contact the physician

   e. Something else. Please specify ____

   ____

3. Have you <u>usually</u> received a patient information sheet concerning your ePrescription(s) from a physician?

1 Yes
2 No, the physician has not given me a patient information sheet go to question **5.**
3 No, because I haven't wanted a patient information sheet go to question **5.**
4 I don't know what a patient information sheet is go to question **5.**

4. In your opinion, was the content of the patient information sheet clear?

1 Yes
2 No. Why not? ____

____

5. How do you check the status of your ePrescription(s)? (e.g., amount of medication remaining or expiry date of a prescription). You may choose several alternatives.

1 I do not check it at all
2 I use the online My Kanta service

3 I ask at the pharmacy
4 I read the label affixed to the medication package
5 I read the patient information sheet
6 I keep track of it myself
7 I use another method. Please specify the method you use ____________________________________

6. Were you told how much medication is remaining on your ePrescription(s) this time at the pharmacy?

1 Yes
2 No

7. Have you renewed your ePrescription(s) through the pharmacy?

1 Yes

2 No □ go to question **9.**

8. How well has renewing your ePrescription(s) through the pharmacy succeeded?

1 There have been no problems in renewing
2 There have been problems in renewing. Please specify ______________________________________

____

9. Have you purchased medication with an ePrescription for another person? (e.g., a child, spouse or other family member)

1 Yes
2 No □ go to question **11.**

10. Have you succeeded in taking care of an ePrescription for another person?

1 Yes
2 No. What kinds of problems have you encountered? ____

____

11. From whom/where have you learned about ePrescriptions? You may choose several alternatives.

1 From no-one/nowhere □ go to question **14.**
2 A nurse
3 A physician
4 A receptionist (at a health centre, medical clinic, hospital)
5 Pharmacy staff
6 A relative/friend
7 The media (TV, radio, newspaper)
8 On the Internet
9 A brochure
10 Somewhere else. Please specify where ____

12. What did you learn about? Circle all the alternatives that apply.

1 The benefits of ePrescriptions for customers
2 Where ePrescriptions are stored
3 From where and how I can purchase my medication prescribed with an ePrescription

4 How another person can purchase my medication prescribed with an ePrescription for me
5 How I can check my prescription information on a computer
6 Who can view my ePrescription information
7 For what purposes my ePrescription information can be used
8 How my ePrescription information is protected against misuse
9 My right to limit the viewing of my ePrescription information
10 Which authorities arrange services related to ePrescriptions

13. Do you feel you have received <u>sufficient</u> information about ePrescriptions?

1 Yes
2 No. What more information would you like? ____________________

____

14. What is your opinion on the following statements? Circle the most suitable alternative for each statement.

	I fully agree	I agree to some extent	I disagree to some extent	I fully disagree	I don't know
It is safe to use ePrescriptions	1	2	3	4	5
It is important for a physician to be able to see what medication other physicians have prescribed for me with ePrescriptions	1	2	3	4	5
It is important for a pharmacy's pharmacist to be able to see all medications prescribed for me with ePrescriptions	1	2	3	4	5
I'm afraid an unauthorized person may view or use my prescription information	1	2	3	4	5
I'm afraid my ePrescription information may be misused	1	2	3	4	5

15. What <u>benefits have you had</u> from using ePrescriptions compared with paper prescriptions?

____

____

____

16. What <u>problems have you had</u> in using ePrescriptions compared with paper prescriptions?

____

____

____

The *My Kanta service* is a Finnish online service intended for adult customers (www.kanta.fi/omakanta), where it is possible to check one's own personal prescription and patient information by logging on with a bank code or other form of identity verification.

17. Are you familiar with the My Kanta service?

1 Yes
2 No go to question **21.**

18. Have you used My Kanta to <u>view your ePrescription information</u>?

1 Yes

2 No go to question **21.**

3

19. Have you printed out a <u>summary of your ePrescriptions</u> from My Kanta?

1 Yes

2 No

20. What is your opinion on the following statements? Circle the most suitable alternative for each statement.

	I fully agree	I agree to some extent	I disagree to some extent	I fully disagree	I don't know
It is easy to log on to My Kanta	1	2	3	4	5
The My Kanta pages are clear and understandable	1	2	3	4	5
It is easy to check the amount of medication remaining on a prescription with My Kanta	1	2	3	4	5
It is easy to check the expiry of a prescription with My Kanta	1	2	3	4	5
It is easy to check if my prescription was renewed with My Kanta	1	2	3	4	5
With My Kanta it is easy to see at which pharmacies and/or healthcare units my ePrescriptions have been viewed	1	2	3	4	5
My Kanta provides a good overall picture of the medications prescribed for me	1	2	3	4	5
My Kanta works without any problems	1	2	3	4	5

21. How satisfied are you with ePrescriptions as a whole? Circle the most suitable alternative.

Not at all satisfied 1 2 3 4 5 6 Very satisfied

22. Your gender?

1 Male
2 Female

23. Your year of birth? 19_____

24. Where do you live?

1 Southern Finland
2 Southwestern Finland
3 Western or Central Finland
4 Eastern Finland
5 Northern Finland
6 Lapland

25. Your education?

1 Basic education (comprehensive school)
2 Vocational degree
3 Upper secondary school graduate
4 Lower-level university degree
5 Higher-level university degree

26. Are you <u>currently</u> using

1 <u>Prescription</u> medication regularly (e.g., high blood pressure medication)
2 <u>Prescription</u> medication only temporarily (e.g., antibiotic, painkiller)
3 Both

You can write any comments you have about this questionnaire and your experiences with ePrescriptions and the My Kanta service in the space below. If necessary, you can also write on the back of the cover letter and return it together with this questionnaire form.

Thank you!
